# The source effect as a natural function of disgust in interpersonal context and its impairment in mental disorders

**DOI:** 10.1038/s41598-019-40802-4

**Published:** 2019-03-12

**Authors:** Maria Lenk, Gerhard Ritschel, Marion Abele, Peggy Roever, Julia Schellong, Peter Joraschky, Kerstin Weidner, Ilona Croy

**Affiliations:** Department of Psychotherapy and Psychosomatic Medicine, University Hospital Carl Gustav Carus Dresden, Technische Universität Dresden, Dresden, Germany

## Abstract

Disgust affects interpersonal relationships and regulates hygienic, sexual and distance behaviour. Its intensity in the interpersonal context depends on the character of the relationship. Strangers normally evoke more disgust than intimates (known as the source effect). General disgust sensitivity is increased in various mental diseases. It is unclear how disgust in the interpersonal context is affected and whether the source effect is preserved. 460 inpatients with mental disorders and 463 healthy subjects answered a newly developed Questionnaire (DIRQ) that covers disgust in the interpersonal context on content categories (hygiene, physical proximity, sexuality) and on source categories (self, partner, parent, stranger). Mental disorders were diagnosed with structured interviews. Healthy controls exhibited a pronounced source effect, with strangers evoking more disgust than intimates. In patients, this source effect was reduced (Cohen’s *d* = 0.3), especially for sexual disgust, while general disgust sensitivity was increased (*d* = 0.5). High disgust in patients was best predicted by a history of sexual abuse and by the presence of post-traumatic stress disorder. In conclusion, mentally impaired patients show increased and trauma-associated disgust sensitivity. Their downregulation of sexual disgust in intimate relationships is hindered, which may have a boundary protective function but might also fuel difficulties engaging in relationships or intimacy.

## Introduction

**Figure 1:**
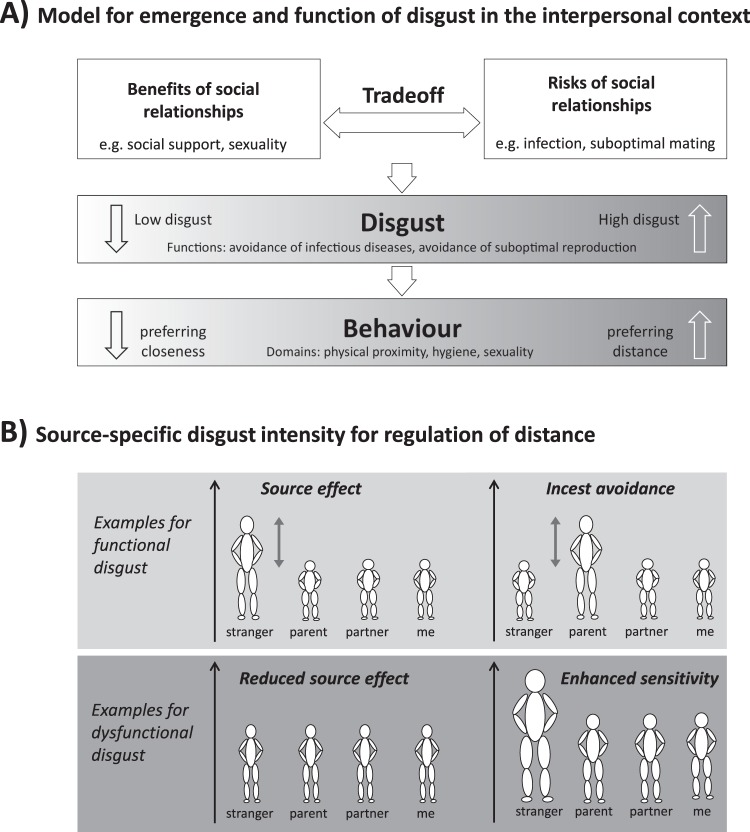
Theoretical model of function and dysfunction of disgust in the interpersonal context. (**A**) Disgust serves the avoidance of disease and suboptimal reproduction. In the interpersonal context, disgust regulates our contact behaviour. To engage in physical contact and intimacy, tradeoffs between benefits and risks must be made. If the risks outweigh the benefits, disgust emerges, and, in consequence, individuals react with avoidance behaviour. (**B**) The amount of disgust in the interpersonal context depends on the evoking subject. Strangers usually elicit more disgust than intimates in order to prevent the entry of unknown pathogens. This so-called source effect serves the avoidance of disease. In the domain of sexual disgust, this source effect is supplemented by mechanisms for the optimization of reproduction, such as incest avoidance. Dysfunctions can manifest in different ways. As examples, the general disgust sensitivity or the source effect could be altered.

When interacting with other people, we have an intuitive sense of the comfortable distance in regard to our counterpart. This sense of distance is regulated by different emotions and cognitive strategies. One aversive emotion regulating distance behaviour is disgust^1^. From an evolutionary point of view, disgust protects from transmission of infectious diseases and suboptimal reproduction^2–6^. It impacts our hygienic, sexual, and contact behaviour. The questions of with whom to come in contact and with whom to have sex are seen as central adaptive problems addressed by disgust and governed by trade-offs between risks and benefits^1^ (compare Fig. 1A).

## The Function of Disgust As a Disease Avoidance Mechanism

Distancing oneself from and avoiding contact with disgusting objects or individuals are characteristic forms of behaviour that represent the purpose of disgust as a mechanism for the avoidance of infectious diseases^2–5^. It has been shown that people who are very sensitive towards disgust and contamination are less often affected by infections^3^ (however, contradictory results also exist, e.g.^7^). Disgust serves as a ‘behavioural immune system’^3,5^ that protects the individual and the group from contamination by pathogens and shapes individual and group hygiene behaviour^5^. As most microbial threats are not directly detectable by human senses, this behavioural immune system is not sensitive to pathogens per se. Instead, disgust is directed against cues that signal that infection risk might be present, whether due to its morphology or olfactory cues that signal the threatening danger of contamination^4^. There is a high concordance between stimuli indicating infectious diseases and stimuli evoking disgust, and therefore, disgust-evoking stimuli occur quite frequently along the transmission paths of infections^4^. Microbial threats enter the body not only via oral ingestion but also through the nose, skin, or sexual organs^8^. Other people’s shortcomings in hygiene may evoke revulsion, especially when related to contact with highly contagious substances such as bodily fluids, blood, or wound secretions^4,8^. Body contact and any exchange of bodily fluids, e.g., through sexual contact, are associated with the danger of contamination by infectious diseases^4,8^.

However, it would not be functional to avoid all interpersonal contacts, as social relationships and intimacy usually bring numerous advantages^1^. Pathogens from outside a social group and are unknown to our immune system constitute a potentially higher risk compared to mutual pathogen strains we share with the people surrounding us^9,10^. Therefore, contacts with strangers might be riskier than contacts with intimates. Trade-offs between infection risks and the benefits of social contact and intimacy are necessary^1^. It would thus seem reasonable that disgust intensity and avoidance depend on the level of familiarity with the other person. Strangers usually evoke more disgust than close relatives. This phenomenon is known as the source effect^8–10^ (compare Fig. 1B).

The source effect has been acknowledged in several studies with different methodologies. For instance, it has been shown that the bodily fluids of strangers evoke more disgust than bodily fluids of intimates^8^, the body odours of strangers evoke more disgust than one’s own body odour^9^, and mothers are more disgusted by the faeces of strangers’ babies than by those of their own newborns^11^. Disgusting stimuli from strangers evoke not only more intense disgust but also more pronounced heart rate reduction and faster avoidance behaviour than stimuli from intimates^10^.

The source effect as a differentiated disgust reaction is an important factor in the moderation of social relationships. Disgust boundaries need to be lowered in social contexts (e.g., shaking hands, sitting on the bus, etc.). Particularly in intimate relationships, this downregulation is essential in order to experience and enjoy caresses, physical proximity, and sexuality. If the source effect is disturbed, the differentiation between intimates and non-intimates may be hindered, and dysfunctional barriers of disgust may emerge in families or partnerships.

## Sexual Disgust and Avoidance of Suboptimal Mating

The intensity of disgust depends on the type of relation with our counterparts and depends on the context^10^. For instance, disgust can be temporarily suppressed in order to give in to stronger drives, such as the satisfaction of hunger, thirst, or sexual desire^4,12^. In addition to mere protection against contamination, sexual disgust seems to have another evolutionary value, namely, the optimization of reproduction^1,6^. Many deviant and disgust-evoking sexual forms of behaviour reduce fitness, which suggests disgust to be an inhibitor of biologically suboptimal sexual behaviour^13^. Biologically undesirable sexual partners, such as alien species, close relatives, children, or very old people, are usually avoided^6^.

Incest is one of the taboos that elicits the strongest disgust^14,15^. Among all incestuous relationships, sexual contacts between parents and their biological children evoke the highest disgust^15^ and are seen as immoral. Within the domain of moral disgust, incest is one of the strongest disgust elicitors^16^.

## From Function to Dysfunction

In summary, many of our attitudes to morality, sexual taboos, and socially desired behaviour, be it behaviour of hygiene or distance, serve the purpose of disgust avoidance. Functional disgust is a central emotion for the regulation of distance behaviour. We assume that disgust can be disturbed in different ways: the overall level of disgust as well as the source effect can be altered (compare Fig. 1B). However, this aspect has been mostly neglected. Current research focuses rather on differences in overall disgust intensity. It is known that increased disgust is related to a variety of mental disorders.

### Mental disorders with close relation to disgust

Some specific phobias and obsessive compulsive disorders are classical disgust-related disorders with a generally increased sensitivity to disgust (compare^2,17–19^) (Note: in the article, the term disgust sensitivity is used for both sensitivity and propensity). Phobias such as blood-injury-injection phobia or small animal phobia are based not only on fear but also on disgust^19^. The dysfunctional cognitions of those phobias are related to classical disgust elicitors such as the destruction of physical integrity or small animals associated with the transmission of infectious diseases (rats, insects), slime or faeces (slugs, snakes)^2^. Moreover, obsessive washing is related to enhanced fear of contamination, which results in unreasonable washing behaviour and in the avoidance of situations with the potential risk of contamination^17^.

### Disgust as a central moderator of interpersonal disorders

In addition to the previously mentioned disorders, which are closely related to disgust-evoking objects, other mental disorders are connected to disgust, although the association seems to be more complex. Enhanced levels of disgust have been described in eating disorders^20–22^, borderline personality disorder^23^, and posttraumatic stress disorder [PTSD]^23,24^. Those disorders are often related to difficulties in interpersonal interactions and relationships and are more frequent in people with early adverse relationship experiences.

Impairments of relationship, affect, and self-regulation are frequent in traumatized patients, and these impairments have immanent consequences for daily life functionality, such as an attitude of mistrust towards others and difficulties engaging in close relationships^25^. Patients with complex PTSD have often been exposed to severe and continuing traumatization during childhood. Growing up in broken homes with sexual and physical abuse and a lack of emotional support shapes emotional development, especially during vulnerable periods of childhood. Therefore, the diversity of complex PTSD symptoms may reflect an individual’s attempt to cope with regulation deficits and adapt to an insecure environment of permanent threat^25^. Against the background of experienced repetitive violation of intimate boundaries in early relationships, closeness and intimacy might be considered as riskier, which fuels aversive emotions such as disgust, shame or anxiety as well as avoidance behaviour.

As disgust is a central emotion of many mental disorders and can appear as a barrier in the therapeutic intervention, it seems important to gain a better understanding of the functions and dysfunctions of disgust in relationships. Therefore, the aim of this study was to investigate disgust in an interpersonal context and its association with relationship distance. We compared disgust in healthy individuals and in patients with mental disorders in order to shed light on potential dysfunctions such as changes in the overall level of disgust and the source effect.

We examined the following hypotheses:

i. Functional disgust as a disease avoidance mechanism might be related to the relationship distance, hence we hypothesize that a source effect can be observed so that disgust towards strangers is more pronounced than disgust towards intimates or the self.
ii. It is believed that disgust also serves the prevention of suboptimal reproduction. Therefore, we hypothesize that in the domain of sexual disgust, the source effect is supplemented by incest avoidance, so that the sexuality of one’s parents evokes more disgust than the sexuality of other sources.
iii. Patients with mental disorders exhibit increased disgust sensitivity and a reduced source effect, hence their disgust experiences are more intense and less selective.
iv. A recalled history of childhood abuse is related to increased disgust in interpersonal contexts in adulthood.

## Material and Methods

### Participants

A total of 923 participants answered the Disgust in Relationship Questionnaire (German version), of whom 463 were healthy participants (aged from 18 to 69 years, ♀ = 64.6% (*n* = 299), ♂ = 35.4% (*n* = 164), mean 28.0 ± 10.6 years), and 460 were inpatients of psychotherapeutic treatment (aged from 18 to 78 years, ♀ = 69.1% (*n* = 318), ♂ = 30.9% (*n* = 142); mean 37.8 ± 12.3 years). Being above the age of 18 and sufficient fluency in the German language served as inclusion criteria, while disordered thinking and psychotic disorders were exclusion criteria. The study was conducted according to the Declaration of Helsinki and approved by the local ethical board (granting body: Ethikkommission an der Technischen Universität Dresden). All methods were carried out in accordance with the guidelines and regulations, and all participants gave written informed consent.

The healthy control group comprised students, hospital staff, patients waiting for a gynaecological routine check, and acquaintances of the researchers. The participants were not compensated for their participation. Patients were recruited within the Department of Psychotherapy and Psychosomatic Medicine of the University Hospital of Dresden (Germany) and represent a typical cross-section of patients within German psychosomatic hospitals with a broad spectrum of diagnoses (diagnosis groups and frequency distribution of diagnoses are shown in Table 1). As our clinic is specialized in the treatment of complex posttraumatic disorders, the sample allows us to specifically investigate patients with traumatic experiences. Among the sample, 111 patients were diagnosed with PTSD, and the majority of those patients were characterized by a complex symptomatology in the context of severe abuse or neglect in childhood or adolescence. According to the classification of severity^26^, the average Childhood Trauma Questionnaire score in this group corresponded to “severe to severest” traumatic abuse (sexual abuse: $\bar{x}=17.76\,({\rm{standard}}\,{\rm{deviation}}\,[SD]=5.955)$; emotional abuse $\bar{x}=13.37\,(SD=7.361)$; physical abuse $\bar{x}=13.70\,(SD=8.373)$). Healthy controls and patients differed in age (*t*[883] = −12.9; *p* < 0.001) but not in sex distribution.

**Table 1:** In the left part of the table the frequency (in percent) of diagnoses in the patient group is reported.

Diagnoses in the patients group		High versus low to moderate disgust sensitivity			
ICD-10 diagnoses	Frequency of Diagnoses in Patients % (n)	Frequency of high disgust within Diagnosis groups %	Regression analysis		
			p	Odds ratio (95% CI)	β
Psychoactive substance use (F10–19)	11.5% (53)	15.1%	0.842	1.098 (0.438 to 2.754)	0.094
Mood disorders (F30–39)	57.4% (264)	12.5%	0.020	0.433 (0.214 to 0.877)	−0.837
Anxiety disorders (F40–41)	40.2% (185)	15.7%	0.662	1.158 (0.599 to 2.240)	0.147
Obsessive-compulsive disorder (F42)	9.1% (42)	21.4%	0.504	1.369 (0.545 to 3.436)	0.314
Post-traumatic stress disorder (F43.1)	24.1% (111)	26.4%	<0.001	4.733 (2.376 to 9.429)	1.555
Dissociative disorders (F44)	3.5% (16)	20.0%	0.987	1.012 (0.237 to 4.324)	0.012
Somatoform disorders (F45)	21.7% (100)	15.0%	0.719	1.139 (0.560 to 2.318)	0.130
Eating disorders (F50)	14.1% (67)	23.9%	0.005	2.776 (1.353 to 5.697)	1.021

The right part of the table contains the frequency (in percent) of high disgust (defined as disgust ≥2.85, compare Fig. 4) within diagnosis groups and results of the logistic regression analysis with the disgust categories ‘high’/’low to moderate’ as dependent variable and diagnoses as independent variables.

### Methods

The Disgust in Relationship Questionnaire [DIRQ] is a newly developed questionnaire that was specifically designed for the purpose of this study. It comprises 44 statements about disgust, which are answered on a 4-point scale (1 – “not true at all” to 4 – “always true”). Each item is built upon two independent dimensions: disgust content and disgust source. The three disgust content categories (hygiene, physical proximity, sexuality) are paired with the four disgust source categories (stranger, parent, partner, self). Every item is accordingly formulated for the four types of relationships. Items about physical proximity are an exception to this rule and are only presented for three disgust sources (stranger, parent, partner), as a second person is necessary for physical proximity.

For the German version of the DIRQ, good internal consistencies for the content scales (Cronbach’s *α* = 0.7–0.9) and good to acceptable internal consistencies for the source categories (*α* = 0.6–0.8) have been found. The DIRQ showed a good convergent validity with the FEE^27^, another questionnaire measuring disgust sensitivity (*r* = 0.729; *p* < 0.001), and good discriminative validity against the Beck Depression Inventory [BDI II]^28^, (German version)^29^, which measures depressive symptomatology (*r* = 0.323; *p* < 0.001). The factorial validity was determined by several factor analyses, which confirmed the three content scales and the source categories within the content scales of sexuality (except item 8) and physical proximity. However, the source structure could not be confirmed within the hygiene subscale. The development of the DIRQ, the psychometric properties as well as the questionnaire items are displayed in the Supplementary Information.

In addition, all patients underwent the structured clinical interview for DSM-IV disorders^30^ for a diagnostic of mental disorders. Furthermore, retrospective information about childhood maltreatment was available for a subgroup of 263 patients. Those patients filled out the reliable and valid Childhood Trauma Questionnaire [CTQ]^31^, (German version)^32^.

### Statistical analysis

Data were analysed with SPSS 24 (IBM Corp., Armonk, NY). For each participant, item scores were averaged per content and source scale, resulting in 11 disgust variables per individual (sexuality: self, partner, parent, stranger; hygiene: self, partner, parent, stranger; proximity: partner, parent, stranger).

We computed several generalized linear mixed models [GLM] (using robust covariances, degrees of freedom adjusted using Satterthwaite approximation), which are described below. Post hoc tests (t-tests) were computed pairwise and adjusted for multiple testing by use of sequential Bonferroni correction. As measure of effect size, we present Cohen’s d with 95% Confidence Interval [95% CI]. The level of significance was set to *α* = 0.05, and all tests were performed as two-sided tests.

First, disgust perception was determined in healthy controls. To this end, a GLM was computed, in which each individual served as a subject, and each of the 11 possible disgust sources by content combinations served as repeated measurement (4 sources for hygiene and sexual disgust; 3 for physical proximity). The disgust value per combination served as the target, and the following fixed effects were computed: main effects of disgust source (4) and gender (2); interaction effects of disgust source (4) by disgust content (3), disgust source (4) by gender (2), and disgust content (3) by gender (2). As age was found to moderately correlate with disgust, it was included in the model as an additional predictor. Second, disgust perception was determined in patients using an identical GLM approach.

In a third GLM, patients were compared to healthy controls. For this GLM, the setup was identical as before and the following fixed effects were computed: main effect of group (2) and interaction effects of disgust source (4) by group (2), disgust content (3) by group (2) and disgust source (4) by disgust content (3) by group (2). Age was added as an additional predictor, as it was correlated to disgust and both groups differed in age.

To capture the source effect more comprehensively, we calculated disgust relative to strangers [RD = relative disgust] as a new variable (e.g., *RD*_*self*_ = (*disgust*_*self*_ − *disgust*_*stranger*_)/*disgust*_*stranger*_). The relative disgust was compared between patients and healthy controls by use of another GLM in which each individual served as the subject and each of the possible combinations of disgust source by disgust content served as the repeated measurement. The absolute value of the RD per combination served as the target and the following fixed effects were computed: main effect of group (2) and interaction effect of disgust content (3) by group (2) and disgust source (3) by group (2). Age was again added as an additional predictor (see above).

The effect of childhood experience on disgust was further examined in the patient group. To this end, each of the 11 disgust variables was correlated with the CTQ subscales using Pearson correlation coefficients. Patients were divided into a high and a low-to-moderate disgust group (cutoff 2.85, compare third subchapter in the result part). Two logistic regression models were performed in order to explain the highly disgust-sensitive subgroup. The CTQ subscales in the first model and the patients’ ICD-10 diagnoses in the second model functioned as regressors. In addition to the standardized regression coefficients *β*, we report odds ratios [OR].

Data relevant to the results are available to other researchers on reasonable request.

## Results

### Disgust and source

#### Disgust and the source effect of disgust in healthy people

**Figure 2:**
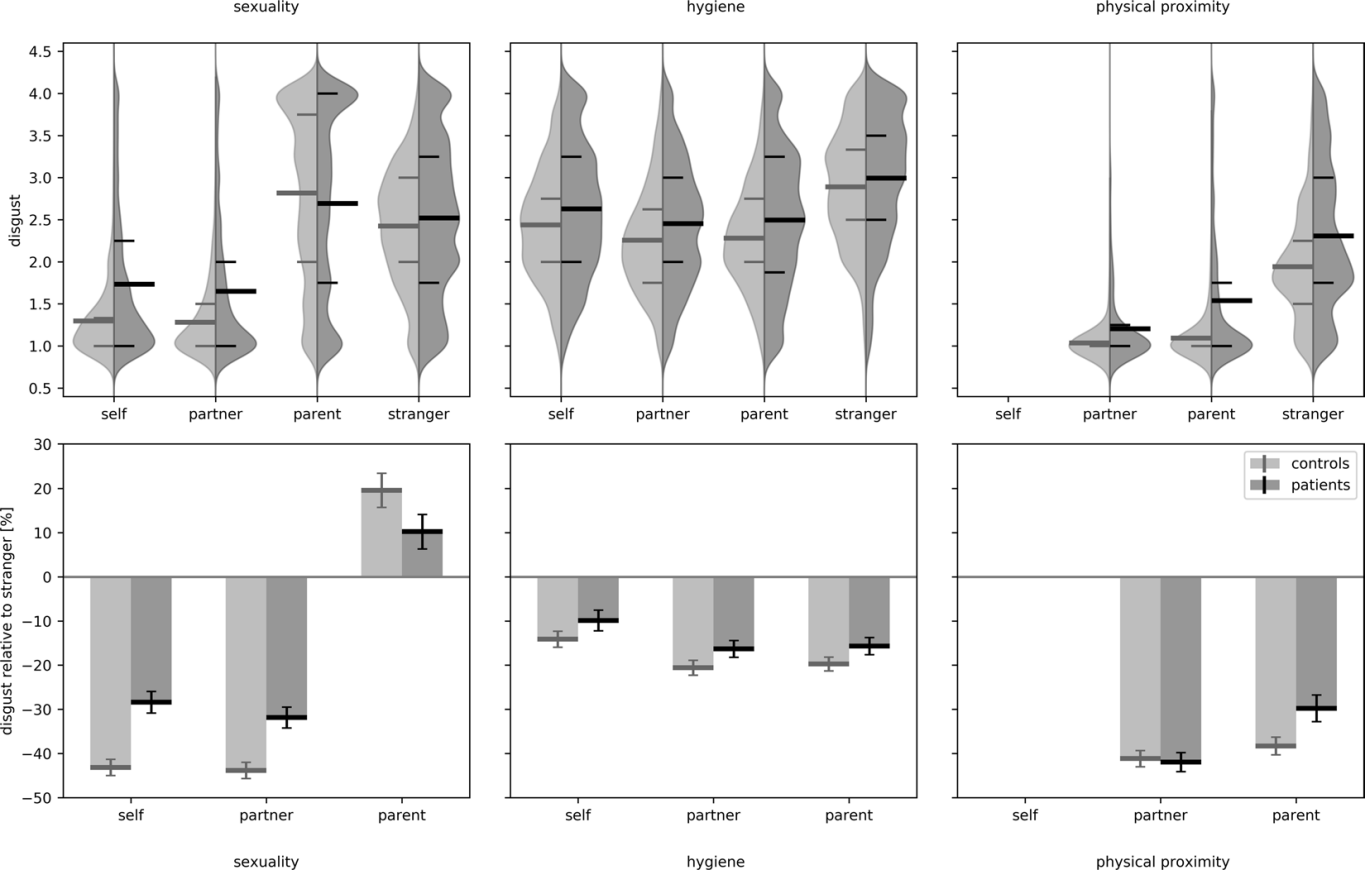
Disgust is increased and source effect is reduced in patients compared to controls. Upper row: disgust for the three content scales ‘sexuality’, ‘hygiene’, and ‘physical proximity’ (from left to right). For each of the content scales, the probability distribution is depicted for the four disgust source scales ‘self’, ‘partner’, ‘parent’, and ‘stranger’ and for the two different groups ‘controls’ (left, light grey) and ‘patients’ (right, dark grey). Additionally, the means (bold lines) as well as the 25^th^ and the 75^th^ percentile (narrow lines) of the distributions are shown. Lower row: relative disgust (as percentage) for the source categories ‘self’, ‘partner’, and ‘parent’ in relation to ‘stranger’ on the three content scales. Depicted are group means and 95% confidence intervals for the two different groups ‘controls’ (left, light grey) and ‘patients’ (right, dark grey).

There was a significant effect of source on disgust intensity (*F*[3; 1578] = 708.8; *p* < 0.001), and a significant source by content interaction (*F*[5; 2283] = 256.1; *p* < 0.001). With one exception, for all three contents, strangers elicited more disgust (see Fig. 2) than parents (hyg: *d* = 0.91; 95% CI [0.77, 1.05]; prox: *d* = 1.78; 95% CI [1.631, 1.936]), the partner (hyg: *d* = 0.96; 95% CI [0.82, 1.09]; prox: *d* = 2.00; 95% CI [1.85, 2.16]; sex: *d* = 2.08; 95% CI [1.91, 2.23]), and oneself (hyg: *d* = 0.65; 95% CI [0.52, 0.78]; sex: *d* = 2.02; 95% CI [1.86, 2.18]). Only the sexuality of one’s parents provoked more disgust than strangers’ sexuality (*d* = −0.51; 95% CI [0.38, 0.64]). All presented comparisons are significant with *p* < 0.001. Analysis of age and gender revealed a slight reduction of disgust with age (*b* = −0.006; *r* = −0.205; *p* < 0.001) and a generally increased disgust level in women (*F*[1; 245] = 25.0; *p* < 0.001; *d* = 0.32; 95% CI [0.19; 0.45]).

#### Disgust and the source effect of disgust in patients with mental disorders

Similar to the healthy controls, there was a significant effect of source on disgust intensity (*F*[3; 2270] = 323.1; *p* < 0.001) and a significant source by content interaction (*F*[5; 3538] = 119.5; *p* < 0.001). Post hoc testing revealed similar behaviour as for healthy controls but smaller effects: Strangers elicited more disgust (see Fig. 2) than parents (hyg: *d* = 0.55; 95% CI [0.42; 0.69]; prox: *d* = 0.90; 95% CI [0.76; 1.03]), the partner (hyg: *d* = 0.62; 95% CI [0.49; 0.75]; prox: *d* = 1.63; 95% CI [1.48; 1.78]; sex: *d* = 1.02; 95% CI [0.88; 1.16]), and oneself (hyg: *d* = 0.43; 95% CI [0.23; 0.56]; sex: *d* = 0.92; 95% CI [0.78; 1.05]). Again, only the sexuality of one’s parents provoked more disgust than strangers’ sexuality (*d* = 0.21; 95% CI [0.08; 0.34]). All presented comparisons are significant with *p* < 0.001.

### Comparison of overall disgust and source disgust between healthy individuals and patients

Patients reported generally increased disgust compared to healthy individuals (*F*[1, 255] = 66.3; *p* < 0.001; *d* = 0.50; 95% CI [0.37; 0.63]). This increased disgust in patients was found for each of the disgust contents (sexuality: *t*[282] = 5.0; *d* = 0.33; 95% CI [0.20; 0.46] hygiene: *t*[341] = 5.0; *d* = 0.33; 95% CI [0.20; 0.46]; physical proximity: *t*[327] = 11.4; *d* = 0.77; 95% CI [0.64; 0.90]) and for each of the disgust sources (self: *t*[326] = 8.6; *d* = 0.56; 95% CI [0.43; 0.70]; partner: *t*[321] = 9.2; *d* = 0.62; 95% CI [0.49; 0.76]; parent: *t*[351] = 4.8; *d* = 0.32; 95% CI [0.19; 0.45]; stranger *t*[386] = 5.1; *d* = 0.34; 95% CI [0.21; 0.47]). All presented comparisons are significant with *p* < 0.001. Patients reported more disgust towards physical proximity with their partner, parent, and a stranger (all *p* < 0.001); more disgust towards violations of hygiene by themselves, the partner, parent (all *p* < 0.001) and a stranger (*p* = 0.007); and more disgust towards their own, their partner’s (all *p* < 0.001) and a stranger’s sexuality (*p* = 0.041), but we found no difference for the disgust related to the sexuality of the parents (*p* = 0.1).

In contrast to the healthy controls, patients’ ratings showed a significantly reduced source effect (main effect of group on RD: *F*[1,1135] = 17.6; *p* < 0.001; *d* = 0.29; 95% CI [0.16; 0.42]), indicating the patients’ reduced ability to differentiate between strangers and intimates. However, there was a significant group by content interaction (*F*[4,1803] = 312.9; *p* < 0.001), and post hoc tests revealed that group differences in RD were based solely on the sexual disgust scale (*t*[967] = 9.4; *p* < 0.001; *d* = 0.61; 95% CI [0.48; 0.74]; *compare* Fig. 2). For hygiene (*p* = 0.5) as well as for physical proximity (*p* = 0.9), there was no significant difference of source effect between patients and controls.

### Association between childhood traumatization, diagnoses, and disgust in patients

**Figure 3:**
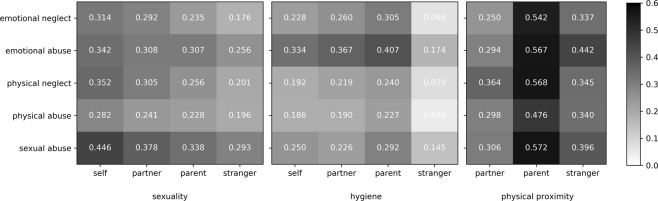
Disgust sensitivity in interpersonal contexts is associated with abuse and neglect in childhood. Correlation coefficients (Pearson) between different forms of childhood maltreatment (measured with the childhood trauma questionnaire, rows) and disgust in interpersonal contexts (columns) are displayed for the patient group.

Abuse and neglect were significantly associated with disgust sensitivity in patients. All CTQ subscales showed positive bivariate correlations with disgust, with the highest correlative connection between disgust and emotional and sexual abuse. In particular, disgust relating to physical proximity of parents was closely related to all forms of childhood traumatization (*compare* Fig. 3). All but the correlations smaller than 0.1 are significant.

**Figure 4:**
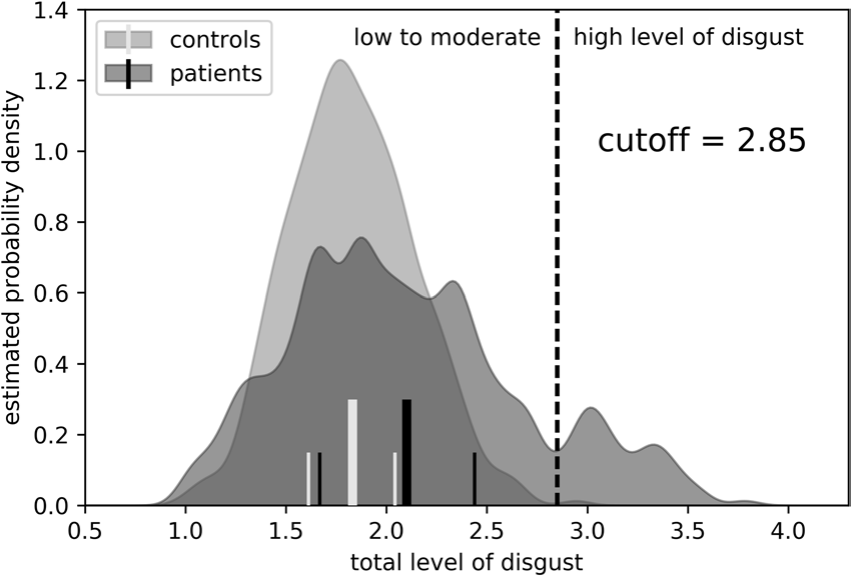
Disgust in patients shows a bimodal distribution. Probability densities for the overall disgust in the two groups ‘controls’ (light grey) and ‘patients’ (dark grey). Additionally depicted are the positions of the means (bold lines) as well as the 25^th^ and the 75^th^ percentile (narrow lines). Within the patient group, the distribution is bimodal, and we therefore separate the subgroup with ‘high disgust’ sensitivity by introducing a cutoff at 2.85. This cutoff is used for a subgroup analysis (see main text).

The graphical depiction of the disgust distribution in Fig. 4 shows a bimodal distribution in the patient sample. Because of this, we introduced a cutoff at 2.85 to separate the two groups from each other. The vast majority of patients (86.9%; *n* = 399) are attributable to the sector of low to moderate disgust, whereas 13.1% (*n* = 60) of the patients belong to a very highly sensitive subgroup. Only one (0.2%) healthy participant was identified as highly disgust-sensitive.

This highly disgust-sensitive subgroup in the patient sample could be partly explained by childhood traumatization and mental disorders. Regression analyses showed that within the different forms of childhood traumatization, the high-disgust subgroup could primarily be explained by sexual abuse (*p* = 0.025; *β* = 0.089; *OR* = 1.093; 95% *CI* = 1.011 to 1.0181), while emotional abuse (*p* = 0.487; *β* = 0.052; *OR* = 1.053; 95% *CI* = 0.910 to 1.218) and neglect (*p* = 0.138; *β* = 0.122; *OR* = 1.129; 95% *CI* = 0.962 to 1.327) as well as physical abuse (*p* = 0.762; *β* = −0.018; *OR* = 0.982; 95% *CI* = 0.874 to 1.104) and neglect (*p* = 0.339; *β* = 0.076; *OR* = 1.079; 95% *CI* = 0.923 to 1.261) had no additional significant impact.

In the second regression model, significant associations between high disgust sensitivity and PTSD as well as eating disorders were identified, whereas patients with mood disorders tended to be low or moderately disgust-sensitive. High disgust sensitivity was most frequent among the patients with PTSD (26.4%) and eating disorders (23.9%; *compare* Table 1).

## Discussion

### Functional disgust underlies a distinct source effect

Our results show a distinct source effect in healthy controls, meaning that disgust stimuli are perceived as more intense if related to a stranger as opposed to intimates or oneself. This effect has been described in different former studies^8–10,33^. Likely, disgust protects the individual and the core group against new unknown pathogens, and the source effect seems to be important to the avoidance of infectious diseases^10^.

In addition to this disease-avoidance mechanism, disgust is believed to serve the avoidance of suboptimal mating^1,6,33^, where incestuous sexuality is one of the strongest disgust-provoking taboos^15,16^. Although we did not ask about incest specifically, incest avoidance becomes apparent in our results. The sexuality of one’s parents evoked more intense disgust than a stranger’s sexuality in both our patients and healthy participants. In addition, the findings of Borg *et al*. gave information about the development of this source-specific sexual disgust. They confirmed that sex-relevant disgust to parents is increased in adolescents compared to preadolescents^33^. This increase of parental sexuality-related disgust with puberty and its high levels in our adult population support the hypothesis of the function of disgust in incest avoidance.

In concordance with recent meta-analyses^34^, our results confirm higher disgust sensitivity in women. The authors explained this gender effect by the benefits of polygynous mating in males, wherefore males might be more willing to take the risk of harm than females^34^.

### Mental diseases are characterized by increased disgust sensitivity and a reduced source effect

Increased levels of disgust have been shown for a variety of mental disorders such as phobias, health anxiety or obsessive compulsive disorders^2,17–19,35^, eating disorders^20–22^, sexual dysfunction^36^, borderline personality disorder^23^, and trauma-related disorders^23,24^. Our patients with mental disorders also showed increased disgust for disgust elicitors in interpersonal contexts. Compared to healthy controls, they were clearly more sensitive to disgust, and we identified a subgroup with even higher disgust sensitivity within the group of all patients with mental disorders. While this subgroup was less prone to depression, which we understand in the context of depression-induced anhedonia, patients with PTSD and eating disorders were clearly overrepresented.

In particular, disturbances in early relationship experiences seem to have long-lasting effects on the disgust system. In our study, all forms of recalled childhood neglect and abuse were related to increased disgust sensitivity. Moreover, it is not only the gravest traumatizing events such as sexual or heavy physical abuse that can be assumed to be pathogenic but also emotional abuse and neglect. Both often occur in the framework of desolate family structures and are characterized by a lack of social and emotional support^25^. Nevertheless, sexual abuse belongs to the severest forms of childhood maltreatment and deeply affects emotional development. In line with this characteristic, sexual abuse was the strongest predictor of high disgust in interpersonal contexts in our study. Analogous to our research, other studies that examined different aspects of disgust confirmed increased disgust in victims of sexual violence. Ille *et al*. investigated self-disgust in patients with mental disorders. They found that those with a history of physical and/or sexual abuse experience higher levels of behavioural disgust and disgust towards their own personality compared to patients without such a history^22^. Several studies examined the relation between PTSD symptoms and disgust using trauma event script techniques^24,37,38^. The symptom load of PTSD related to sexual abuse was found to be associated with the peritraumatic disgust experience as well as with posttraumatic disgust reactivity^24^. The comparison of women with and without a history of sexual abuse shows that sexual abuse is related not only to higher levels of disgust but also to feelings of dirtiness and the urge to wash oneself in reaction to an event script^38^. Olatunji *et al*. could confirm the relation between disgust and the development of PTSD by comparing the disgust experience of traumatized adults who did or did not develop PTSD. They found that women who developed PTSD had a more intense disgust experience than women without this diagnosis^37^.

Early childhood traumatization has a negative impact on the development of secure attachment, stress modulation, and affect regulation^25^. Our results confirm an increased and trauma-associated sensitivity of disgust in patients with mental disorders. Patients’ reduced source effect, especially in the domain of sexual disgust, indicates that the down-regulation of disgust in intimate relationships might be impaired. To engage in intimacy and sexual contacts, the benefits of those actions should outweigh the risks^1^. For people who have been exposed to severe violation of their intimate boundaries and who have experienced closeness and sexuality as threatening, the negative consequences may predominate, and aversive reactions may be strengthened. We hypothesize that via avoidance behaviour and formation of distance, disgust may, on the one hand, serve as a boundary protective mechanism. On the other hand, such pronounced disgust may fuel difficulties engaging in close relationships or intimacy—problems that are well-known in patients with complex PTSD^25^.

### Limitations and outlook

The naturalistic sample of patients represents the typical spectrum of diseases in a German clinic of psychosomatic medicine. The majority of our patients with PTSD had complex traumas and suffered early childhood traumatization. For assessing childhood data retrospectively by self-report, a memory bias cannot be excluded^39^, however, the clinical treatment record aligns well with the self-reports. A replication of our results on a different clientele of patients and in international comparison would be of interest. Controls and patients were not age-matched, and therefore we included age as additional predictor in our model.

Because of the cross-sectional design, our study cannot answer questions about the direction of the shown associations, so interpretations in terms of causality must be considered as hypothetical and would require a longitudinal approach.

To capture the association between disgust intensity and relationship distance, we developed a new instrument in which the sources of disgust in interpersonal relationships are varied systematically. The subscales of the DIRQ show acceptable to good internal consistencies. The factor structure was confirmed for the content scales and the source categories, except for the source categories within the hygiene subscale. The factor structure within the hygiene scale was dominated by the item phrasing. (The phrasing of each item was repeated four times across the questionnaire with variation of the source; see Supplementary Information). The variance related to the different item phrasings was larger than the variance related to the source categories. It cannot be conclusively clarified whether the hygiene source effect was actually slight or whether our item phrasing was too heterogeneous. Therefore, the hygiene source categories should be interpreted with caution until their validity is proven in further studies. However, good validity can be assumed for the content aspect of the hygiene scale.

Although it is well-known that changes in disgust experience are relevant for several mental disorders, disgust is still an often-neglected barrier, also in the therapeutic relationship. A better understanding of its distance regulating function might help to address this barrier in the therapeutic context. We hope that our study will stimulate further research on the impact of disgust in relationships on attachment, the therapeutic process, and the therapy outcome of patients in mental health care.

## Supplementary information

Supplementary Information
