# Supplementary material for: The source effect as a natural function of disgust in interpersonal context and its impairment in mental disorders
Source: Sci Rep. 2019 Mar 12;9:4239. doi: 10.1038/s41598-019-40802-4 (PMC6414517; doi:10.1038/s41598-019-40802-4)
Supplement: Supplementary file 1 — Supplementary Information [file 41598_2019_40802_MOESM1_ESM.pdf]

<u>Supplementary Information</u>

The source effect as a natural function of disgust in interpersonal context and its impairment in mental disorders

Maria Lenk*, Gerhard Ritschel, Marion Abele, Peggy Roever, Julia Schellong, Peter Joraschky, Kerstin Weidner and Ilona Croy

DIRQ

The following gives supplemental information about the “Disgust in Relationship Questionnaire” [DIRQ]. This questionnaire was developed for and used in the study, which is reported in the article “The source effect as a natural function of disgust in interpersonal context and its impairment in mental disorders”.

Development of the questionnaire

The DIRQ was developed in three steps. In the first step of questionnaire development, 152 items were formulated belonging to 5 content categories (hygiene, physical proximity, sexuality, disease and morality) and five source categories (stranger, acquaintance, parent, partner, self). Those items were tested in 66 healthy persons (mean age 29.6 +/-12.5 years, 33 women) for usability and understandability. Items that were mistakable were removed. On the disgust source scale, we observed a high concordance between the categories ‘stranger’ and ‘acquaintance’ and therefore removed the category ‘acquaintance’.

In the second step, the remaining questionnaire, which consisted of 88 items, was presented to 171 healthy persons (mean age: 22.3 +/- 2.3 years, 116 women), most of whom were students. We observed a high concordance between the content categories ‘hygiene’ and ‘disease’, which is why we merged those items under the category ‘hygiene’. Furthermore, the content category ‘morality’ resulted in very heterogeneous answers on the item level, which is why we omitted this category.

In the third step, the remaining questionnaire, which consisted of 52 items, was presented to 271 healthy persons (age: 28.2 +/- 9.8 years, 124 women), after which 8 more items were removed based on a weak correlation with the respective content and source scales.

Structure and psychometric properties of the questionnaire

The German version and the translated English version of the questionnaire with 44 items are presented in Fig. S1. The items refer to the disgust content categories hygiene, physical proximity, and sexuality and to the disgust source categories stranger, parent, partner, and self. All items are answered on a 4-point scale with the anchors 1–'not true at all' and 4–'always true'. As a result, an overall mean score can be computed as well as a mean score per subscale (related items per scale are shown in Tab. S6).

Tests of reliability reveal good internal consistencies for the content scale (physical proximity: Cronbach's $\alpha = 0.864$; sexuality: $\alpha = 0.859$; hygiene: $\alpha = 0.719$) and good-to-acceptable internal consistencies for the source categories (self: $\alpha = 0.637$; partner: $\alpha = 0.643$; parent: $\alpha = 0.778$; stranger: $\alpha = 0.836$). Tests of validity show good convergent validity with the FEE [1], another questionnaire that measures disgust sensitivity ($r = 0.729$; $p < 0.001$), and good discriminative validity against the Beck Depression Inventory [BDI II] [2] (German version [3]), which measures depressive symptomatology ($r = 0.323$; $p < 0.001$).

Because of the dual structure of the questionnaire, with the pairing of both factors disgust source and disgust content in every item, the validity of factor analysis is restricted. However, principle components analysis with Varimax rotation (based on eigenvalues >1) performed in the group of healthy participants (n=463) confirmed the three content categories (results are shown in Tab. S1). Additionally, we performed three factor analyses, one for each content scale, in order to test whether the questionnaire separates the source categories (results are shown in Tab. S2 to S4). Within the content scales of physical proximity and sexuality, the intended factor structure for the source categories could be confirmed with one exception: Item 8 ("I feel disgusted when noticing sperm/vaginal fluid on me.") showed a higher loading on the factor "partner" than on factor "self". However, the source structure could not be confirmed within the hygiene subscale. The factor structure within the hygiene scale was dominated by the item phrasing instead. Descriptive characteristics of the DIRQ in patients and healthy controls are shown in Tab. S5.

Tab. S1:

Factor loadings for exploratory factor analysis with Varimax rotation of DIRQ subscales in healthy controls

	Component		
DIRQ Subscale	1	2	3
Hygiene: stranger	**0.836**		
Hygiene: parent	**0.827**		
Hygiene: partner	**0.817**		
Hygiene: self	**0.793**		
Sexuality: stranger	0.477	**0.578**	
Sexuality: parent	0.503	0.440	
Sexuality: self		**0.855**	
Sexuality: partner		**0.854**	
Physical proximity: stranger	0.504		0.349
Physical proximity: parent			**0.795**
Physical proximity: partner			**0.780**

Note. Only facor loadings > 0.3 are shown. Sum of the squared loadings:
Component 1: 31,4%; Component 2: 50,7%; Component 3: 63,4%

Tab. S2:

Factor loadings for exploratory factor analysis with Varimax rotation for the Items of the content scale sexuality in healthy controls

	Componet			
DIRQ items content scale sexuality	1 parent	2 partner	3 stranger	4 self
I feel disgusted when noticing sperm/vaginal fluid on me.		0.698		
I feel disgusted when I am masturbating.				**0.827**
I feel disgusted when imagining myself having sex.				**0.799**
I feel disgusted by my own sexual fantasies.				**0.541**
I feel disgusted when noticing sperm/vaginal fluid on my partner.		**0.810**		
I feel disgusted by imagining my partner having sex.		**0.692**		
I feel disgusted when noticing my partner masturbating.		**0.544**	0.334	0.308
I feel disgusted when I get to know about my partners sexual fantasies.		**0.435**		
I feel disgusted when I get to know about one of my parents' sexual fantasies.	**0.832**			
I feel disgusted by imagining my parents having sex.	**0.862**			
I feel disgusted when noticing one of my parents masturbating.	**0.788**			
I feel disgusted when noticing sperm/vaginal fluid on one of my parents.	**0.806**			
I feel disgusted when noticing a stranger masturbating.	0.316		**0.748**	
I feel disgusted when I get to know about a stranger's sexual fantasies.			**0.697**	
I feel disgusted when noticing sperm/vaginal fluid on a stranger.	0.417		**0.701**	
I feel disgusted by imagining a stranger having sex.			**0.446**	0.359

Note. Only facor loadings > 0.3 are shown. Sum of the squared loadings:
Component 1: 19.6%; Component 2: 34.1%; Component 3: 38.1%; Component 4: 60.5%

Tab. S3:

Factor loadings for exploratory factor analysis with Varimax rotation for the Items of the content scale physical proximity in healthy controls

DIRQ items content scale physical proximity	Component<br>1<br>stranger	 2<br>parent	 3<br>partner
I feel disgusted when my partner hugs me for greeting.			**0.649**
I feel disgusted when my partner is sitting too close to me in a bus and touches me.			**0.704**
I feel disgusted when my partner is being pushed at me in the elevator.		0.309	**0.450**
I feel disgusted when my partner unintentionally touches me in a crowd.			**0.448**
I feel disgusted when one of my parents hugs me for greeting.		**0.664**	
I feel disgusted when one of my parents is being pushed at me in the elevator.		**0.668**	
I feel disgusted when one of my parents unintentionally touches me in a crowd.		**0.640**	
I feel disgusted when one of my parents is sitting too close to me in a bus and touches me.		**0.742**	
I feel disgusted when a stranger is being pushed at me in the elevator.	**0.861**		
I feel disgusted when a stranger unintentionally touches me in a crowd.	**0.740**		
I feel disgusted when a stranger is sitting too close to me in a bus and touches me.	**0.866**		
I feel disgusted when a stranger hugs me for greeting.	**0.700**		

Note. Only facor loadings > 0.3 are shown. Sum of the squared loadings:
Component 1: 21.9%; Component 2: 40.2%; Component 3: 50.2%

Tab. S4:

Factor loadings for exploratory factor analysis with Varimax rotation for the Items of the content scale hygiene in healthy controls

DIRQ items content scale Hygiene	 1	 2	Componet<br>3	 4
I feel disgusted when I don’t wash my hands after using the toilet.	0.839			
I feel disgusted when I am vomiting.		0.716		
I feel disgusted when seeing a purulent skin disease on myself.			0.739	
I feel disgusted when having bad breath.				0.634
I feel disgusted when noticing my partner not washing his hands after using the toilet.	0.856			
I feel disgusted when noticing my partner vomiting.		0.804	0.312	
I feel disgusted when seeing a purulent skin disease at my partner.			0.867	
I feel disgusted when I become aware of my partner having bad breath.				0.725
I feel disgusted when noticing one of my parents not washing his hands after using the toilet.	0.841			
I feel disgusted when noticing one of my parents vomiting.		0.840		
I feel disgusted when seeing a purulent skin disease at one of my parents.			0.855	
I feel disgusted when I become aware of one of my parents having bad breath.				0.755
I feel disgusted when noticing a stranger not washing his hands after going to the toilet.	0.792			
I feel disgusted when noticing a stranger vomiting.		0.806		
I feel disgusted when seeing a purulent skin disease at a stranger.		0.317	0.620	
I feel disgusted when I become aware of a stranger having bad breath.				0.751

Note. Only facor loadings > 0.3 are shown. Sum of the squared loadings:
Component 1: 18.3%; Component 2: 35.8%; Component 3: 53.0%; Component 4: 67.5%

Tab. S5:

Mean, SD and percentiles in patients and healthy controls

	Healthy					Patients				
DIRQ Subscale	mean	SD	25.	50.	75.	mean	SD	25.	50.	75.
Hygiene: self	2.44	0.64	2.00	2.50	2.75	2.63	0.79	2.00	2.50	3.25
Hygiene: partner	2.26	0.62	1.75	2.25	2.75	2.45	0.75	2.00	2.50	3.00
Hygiene: parent	2.28	0.59	2.00	2.25	2.75	2.50	0.83	1.75	2.50	3.25
Hygiene: stranger	2.89	0.65	2.50	3.00	3.33	2.99	0.76	2.50	3.00	3.50
Sexuality: self	1.30	0.39	1.00	1.25	1.33	1.73	0.86	1.00	1.33	2.25
Sexuality: partner	1.28	0.39	1.00	1.00	1.50	1.65	0.84	1.00	1.25	2.00
Sexuality: parent	2.82	0.98	2.00	3.00	3.75	2.69	1.12	1.75	2.75	4.00
Sexuality: stranger	2.42	0.67	2.00	2.50	3.00	2.52	0.91	1.75	2.50	3.25
Physical proximity: partner	1.04	0.14	1.00	1.00	1.00	1.20	0.44	1.00	1.00	1.25
Physical proximity: parent	1.09	0.23	1.00	1.00	1.00	1.54	0.84	1.00	1.00	1.75
Physical proximity: stranger	1.94	0.61	1.50	2.00	2.25	2.31	0.87	1.75	2.25	3.00

Fig. S1: Disgust In Relationship Questionnaire (DIRQ) – original German version

Bitte beantworten Sie die Fragen ganz spontan. Es gibt keine falschen oder richtigen Antworten, wichtig ist nur Ihr persönliches Empfinden. Einige der aufgeführten Dinge werden Ihnen vielleicht noch nie passiert sein, dann stellen Sie sich bitte vor, in welchem Ausmaß Sie sich ekeln würden.

	Alter:<br>Geschlecht:	**(1) Trifft gar nicht zu**	**(2) Trifft manchmal zu**	**(3) Trifft häufig zu**	**(4) Trifft immer zu**
1	Ich ekle mich, wenn ich mir die Hände nach dem Toilettenbesuch nicht wasche.				
2	Ich ekle mich, wenn ich von den sexuellen Fantasien meines Partners erfahre.				
3	Ich ekle mich, wenn ich bemerke, dass ein Elternteil Mundgeruch hat.				
4	Ich ekle mich, wenn ich mir einen Fremden beim Sex vorstelle.				
5	Ich ekle mich vor meinen sexuellen Fantasien.				
6	Ich ekle mich, wenn ich bemerke, dass sich mein Partner die Hände nach dem Toilettenbesuch nicht wäscht.				
7	Ich ekle mich, wenn ich bemerke, dass ein Fremder Mundgeruch hat.				
8	Ich ekle mich, wenn ich an mir Sperma/Vaginalflüssigkeit bemerke.				
9	Ich ekle mich, wenn ich bemerke, dass sich ein Elternteil die Hände nach dem Toilettenbesuch nicht wäscht.				
10	Ich ekle mich, wenn ich bemerke, wie ein Elternteil sich selbst befriedigt.				
11	Ich ekle mich, wenn ich mich selbst befriedige.				
12	Ich ekle mich, wenn ich an meinem Partner Sperma/Vaginalflüssigkeit bemerke.				
13	Ich ekle mich, wenn ich mir meinen Partner beim Sex vorstelle.				
14	Ich ekle mich, wenn mich ein Fremder in einer Menschenmenge zufällig streift.				
15	Ich ekle mich, wenn ich bemerke, wie mein Partner sich selbst befriedigt.				
16	Ich ekle mich, wenn ich bemerke, dass sich ein Fremder die Hände nach dem Toilettenbesuch nicht wäscht.				
17	Ich ekle mich, wenn mein Partner im Fahrstuhl an mich gedrängt wird.				
18	Ich ekle mich, wenn ein Elternteil im Fahrstuhl an mich gedrängt wird.				
19	Ich ekle mich, wenn ich mir vorstelle, wie ich Sex habe.				
20	Ich ekle mich, wenn mich ein Elternteil in einer Menschenmenge zufällig streift.				
21	Ich ekle mich, wenn ich eine eitrige Hauterkrankung bei einem Elternteil sehe.				
22	Ich ekle mich, wenn mein Partner im Bus zu dicht neben mir sitzt und mich berührt.				
23	Ich ekle mich, wenn ich an einem Elternteil Sperma/Vaginalflüssigkeit bemerke.				
24	Ich ekle mich, wenn ein Elternteil im Bus zu dicht neben mir sitzt und mich berührt.				
25	Ich ekle mich, wenn ich bemerke, dass sich ein Fremder übergibt.				

		(1) Trifft gar nicht zu	(2) Trifft manchmal zu	(3) Trifft häufig zu	(4) Trifft immer zu
26	Ich ekle mich, wenn ich bemerke, wie ein Fremder sich selbst befriedigt.				
27	Ich ekle mich, wenn mich mein Partner bei der Begrüßung umarmt.				
28	Ich ekle mich, wenn ich Mundgeruch habe.				
29	Ich ekle mich, wenn ich von den sexuellen Fantasien von einem Fremden erfahre.				
30	Ich ekle mich, wenn ein Fremder im Fahrstuhl an mich gedrängt wird.				
31	Ich ekle mich, wenn ich eine eitrige Hauterkrankung bei meinem Partner sehe.				
32	Ich ekle mich, wenn mich mein Partner in einer Menschenmenge zufällig streift.				
33	Ich ekle mich, wenn ich bemerke, wie sich ein Elternteil übergibt.				
34	Ich ekle mich, wenn mich ein Elternteil bei der Begrüßung umarmt.				
35	Ich ekle mich, wenn ein Fremder im Bus zu dicht neben mir sitzt und mich berührt.				
36	Ich ekle mich, wenn ich mich übergebe.				
37	Ich ekle mich, wenn ich eine eitrige Hauterkrankung bei mir sehe.				
38	Ich ekle mich, wenn ich bemerke, dass mein Partner Mundgeruch hat.				
39	Ich ekle mich, wenn ich an einem Fremden Sperma/Vaginalflüssigkeit bemerke.				
40	Ich ekle mich, wenn ich bemerke, wie sich mein Partner übergibt.				
41	Ich ekle mich, wenn ich eine eitrige Hauterkrankung bei einem Fremden sehe.				
42	Ich ekle mich, wenn mich ein Fremder bei der Begrüßung umarmt.				
43	Ich ekle mich, wenn ich von den sexuellen Fantasien von einem Elternteil erfahre.				
44	Ich ekle mich, wenn ich mir ein Elternteil beim Sex vorstelle.				

Disgust In Relationship Questionnaire (DIRQ) – English translation

Please answer the questions spontaneously. There are no correct or wrong answers. Only your personal perception is important. Some of the mentioned situations might have possibly never happened to you before. If so, please imagine to what extent you would feel disgusted.

	Age:<br><br>Gender:	(1) not true at all	(2) sometimes true	(3) often true	(4) always true
1	I feel disgusted when I don't wash my hands after using the toilet.				
2	I feel disgusted when I get to know about my partners sexual fantasies.				
3	I feel disgusted when I become aware of one of my parents having bad breath.				
4	I feel disgusted by imagining a stranger having sex.				
5	I feel disgusted by my own sexual fantasies.				
6	I feel disgusted when noticing my partner not washing his hands after using the toilet.				
7	I feel disgusted when I become aware of a stranger having bad breath.				
8	I feel disgusted when noticing sperm/vaginal fluid on me.				
9	I feel disgusted when noticing one of my parents not washing his hands after using the toilet.				
10	I feel disgusted when noticing one of my parents masturbating.				
11	I feel disgusted when I am masturbating.				
12	I feel disgusted when noticing sperm/vaginal fluid on my partner.				
13	I feel disgusted by imagining my partner having sex.				
14	I feel disgusted when a stranger unintentionally touches me in a crowd.				
15	I feel disgusted when noticing my partner masturbating.				
16	I feel disgusted when noticing a stranger not washing his hands after going to the toilet.				
17	I feel disgusted when my partner is being pushed at me in the elevator.				
18	I feel disgusted when one of my parents is being pushed at me in the elevator.				
19	I feel disgusted when imagining myself having sex.				
20	I feel disgusted when one of my parents unintentionally touches me in a crowd.				
21	I feel disgusted when seeing a purulent skin disease at one of my parents.				
22	I feel disgusted when my partner is sitting too close to me in a bus and touches me.				
23	I feel disgusted when noticing sperm/vaginal fluid on one of my parents.				
24	I feel disgusted when one of my parents is sitting too close to me in a bus and touches me.				
25	I feel disgusted when noticing a stranger vomiting.				

		(1) not true at all	(2) sometimes true	(3) often true	(4) always true
26	I feel disgusted when noticing a stranger masturbating.				
27	I feel disgusted when my partner hugs me for greeting.				
28	I feel disgusted when having bad breath.				
29	I feel disgusted when I get to know about a stranger's sexual fantasies.				
30	I feel disgusted when a stranger is being pushed at me in the elevator.				
31	I feel disgusted when seeing a purulent skin disease at my partner.				
32	I feel disgusted when my partner unintentionally touches me in a crowd.				
33	I feel disgusted when noticing one of my parents vomiting.				
34	I feel disgusted when one of my parents hugs me for greeting.				
35	I feel disgusted when a stranger is sitting too close to me in a bus and touches me.				
36	I feel disgusted when I am vomiting.				
37	I feel disgusted when seeing a purulent skin disease on myself.				
38	I feel disgusted when I become aware of my partner having bad breath.				
39	I feel disgusted when noticing sperm/vaginal fluid on a stranger.				
40	I feel disgusted when noticing my partner vomiting.				
41	I feel disgusted when seeing a purulent skin disease at a stranger.				
42	I feel disgusted when a stranger hugs me for greeting.				
43	I feel disgusted when I get to know about one of my parents' sexual fantasies.				
44	I feel disgusted by imagining my parents having sex.				

Tab. S6:

Related items per scale (to obtain mean values, average horizontally for the disgust content scales and vertically for the disgust source scales)

Items disgust content scales	Items disgust source scales			
	Self	Partner	Parent	Stranger
Hygiene	1, 28, 36, 37	6, 31, 38, 40	3, 9, 21, 33	7, 16, 25, 41
Sexuality	5, 8, 11, 19	2, 12, 13, 15	10, 23, 43, 44	4, 26, 29, 39
Physical Proximity		17, 22, 27, 32	18, 20, 24, 34	14, 30, 35, 42

References

1 Schienle, A., Walter, B., Stark, R. & Vaitl, D. Ein Fragebogen zur Erfassung der Ekelempfindlichkeit (FEE). *Zeitschrift für Klinische Psychologie und Psychotherapie* **31**, 110-120, doi:10.1026/0084-5345.31.2.110 (2002).

2 Beck, A., Steer, R. & Brown, G. BDI-II, Beck depression inventory: manual: Psychological Corp. *San Antonio, TX* (1996).

3 Hautzinger, M., Keller, F. & Kühner, C. BDI II–Beck Depressions-Inventar–Manual. *Frankfurt a. M.: Harcourt Test Services* (2006).
